# Supplementary figures and images for: Vulnerability to recurrent episodes of acute decompensation/acute-on-chronic liver failure characterizes those triggered by indeterminate precipitants in patients with liver cirrhosis
Source: PLoS One. 2021 Apr 13;16(4):e0250062. doi: 10.1371/journal.pone.0250062 (PMC8043384; doi:10.1371/journal.pone.0250062)

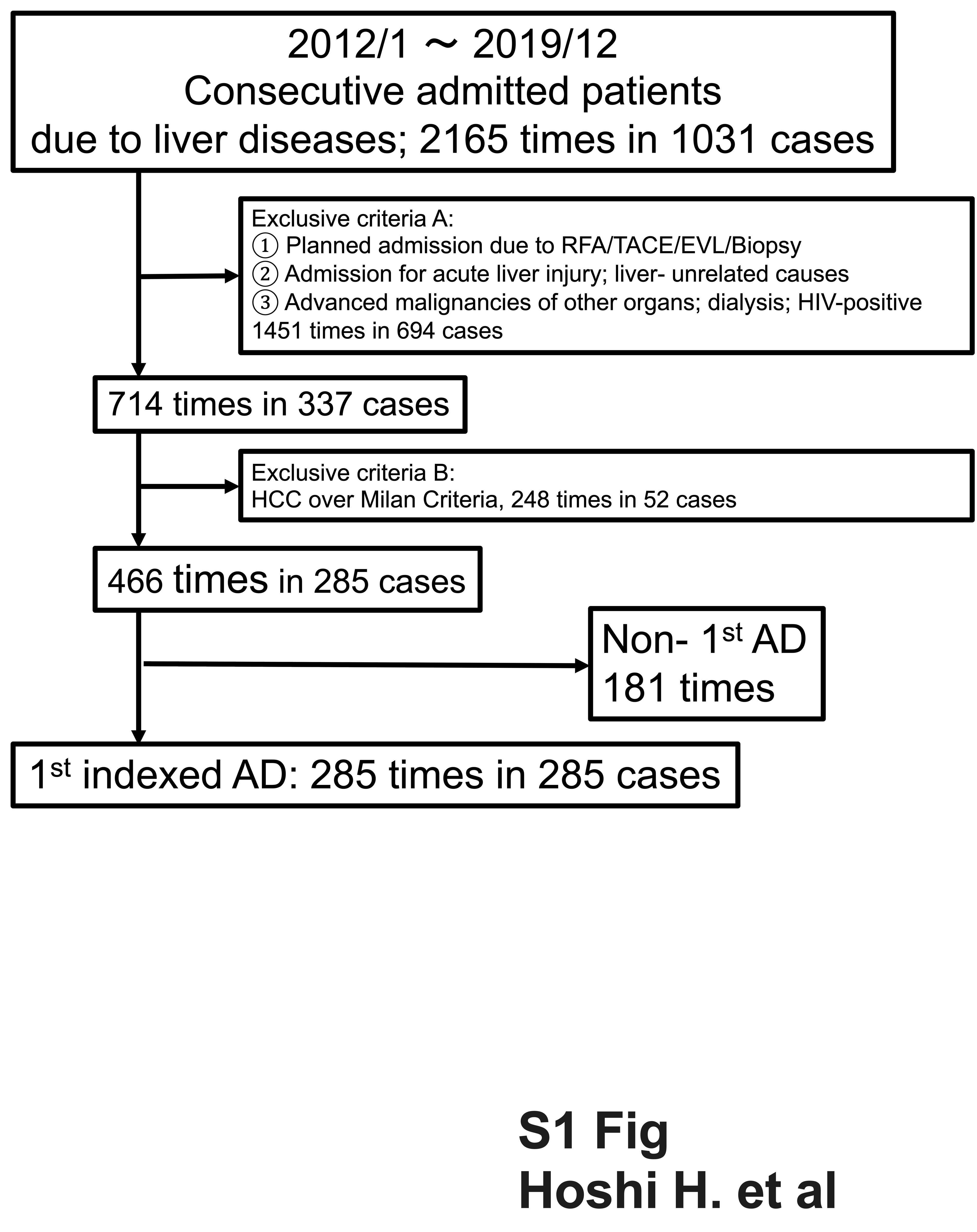

Supplement: S1 Fig — Abbreviations: AD, acute decompensation; RFA, radiofrequency ablation; TACE, transcatheter arterial chemoembolization; EVL, endoscopic variceal ligation; HIV, human immunodeficiency virus; HCC, hepatocellular carcinoma. (TIFF) [file pone.0250062.s002.tiff]

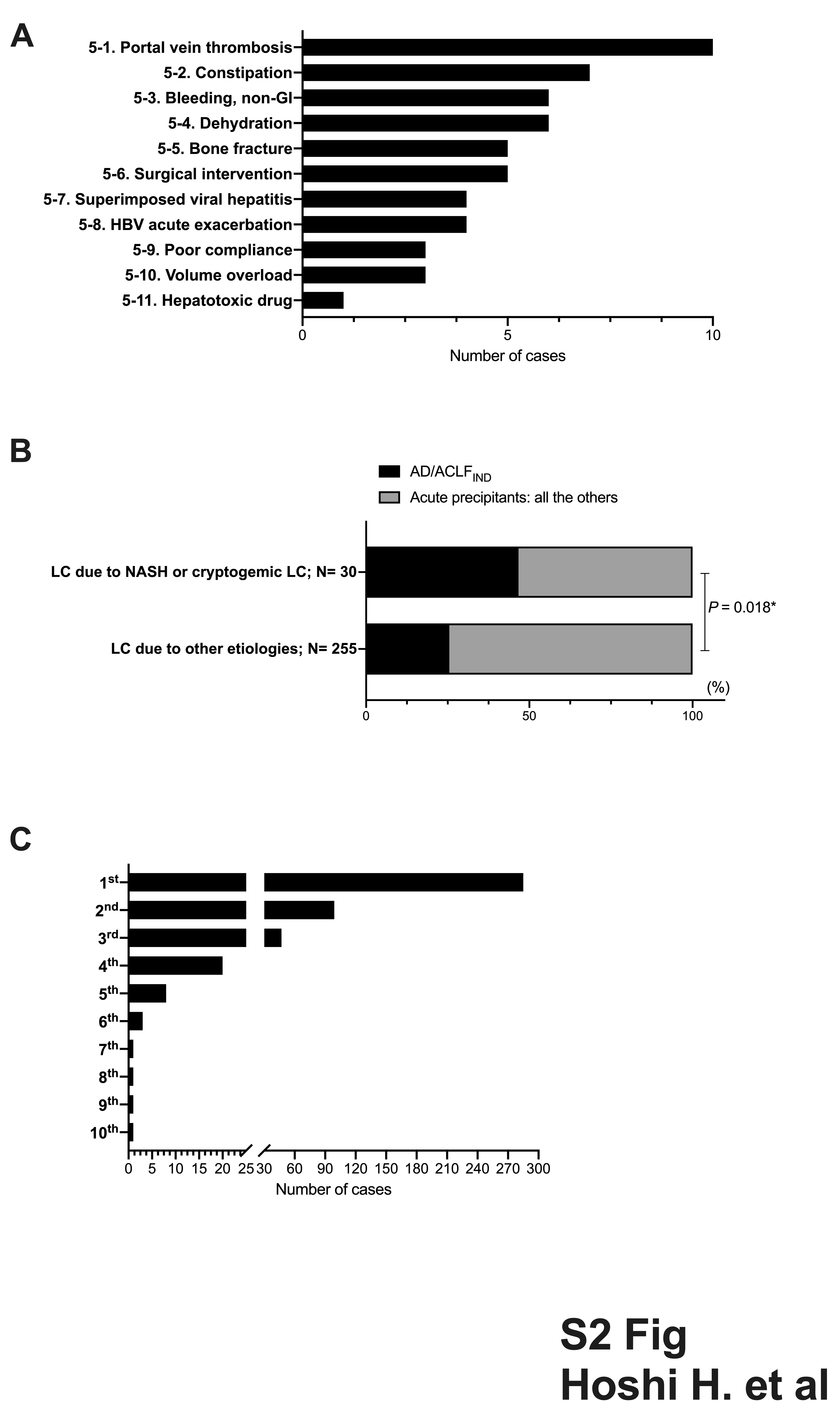

Supplement: S2 Fig — (A) Number of cases and details of miscellaneous causes of acute precipitants for the first indexed AD/ACLF are shown. (B) Percentages of AD/ACLF caused by any indeterminate factor or others were compared by etiologies of LC (NASH and cryptogenic vs others). *P < 0.05. (C) Number of cases from the first to the tenth AD/ACLF observed in the study subjects during the observation period. Abbreviations: AD, acute decompensation; ACLF, acute-on-chronic liver failure; LC, liver cirrhosis; NASH, non-alcoholic steatohepatitis; GI, gastrointestinal; HBV, hepatitis B virus. (TIFF) [file pone.0250062.s003.tiff]

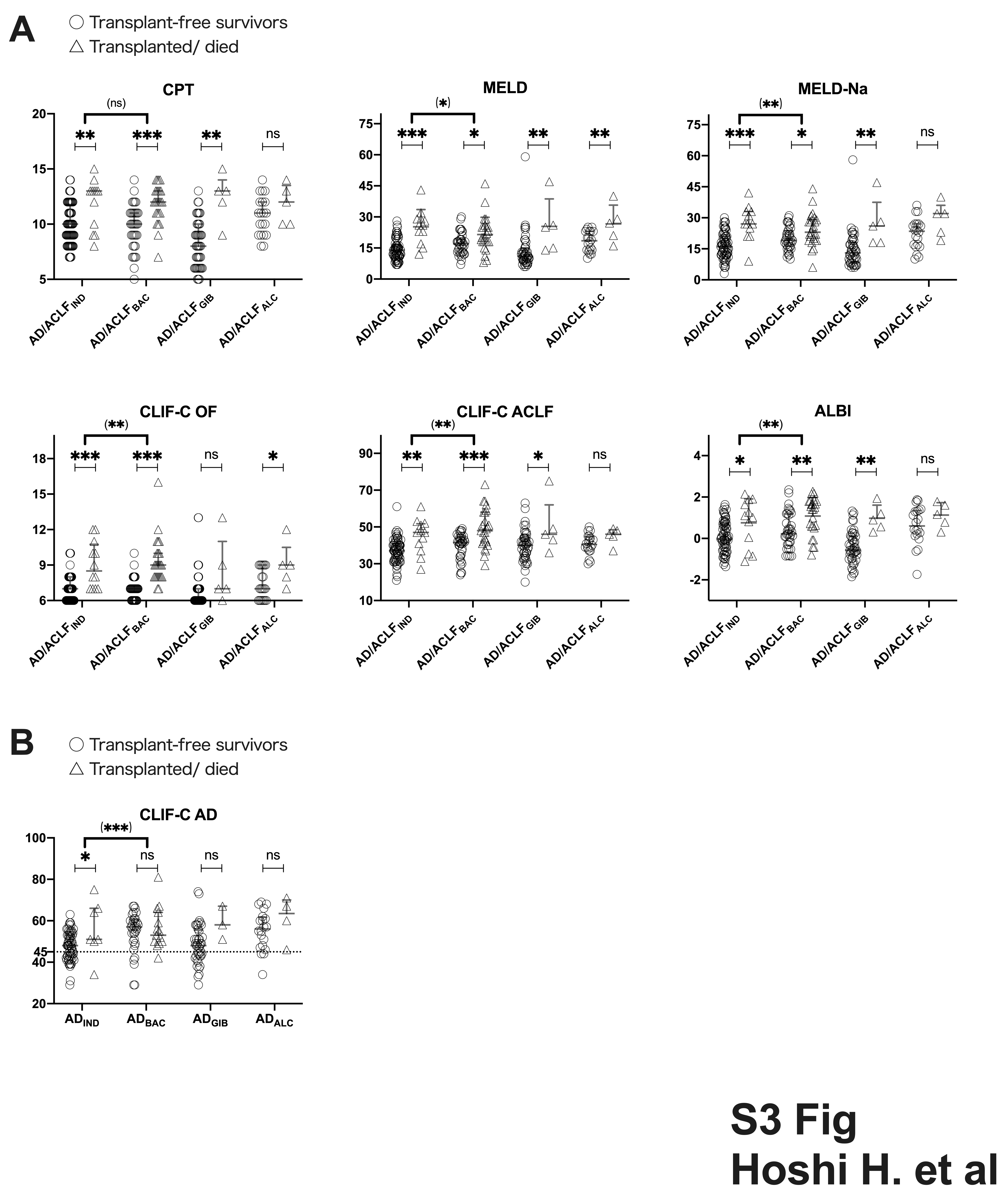

Supplement: S3 Fig — (A) For the first indexed AD/ACLF, various prognostic systems are compared by acute precipitants and outcomes. (B) For the first indexed AD (excluding ACLF grade 1–3), CLIF-C AD scores are compared by acute precipitants and outcomes. Open circles, transplant-free survival; open triangles, liver transplanted/died. P-values within paratheses are for comparison of all patients between indeterminate and bacterial infections. Data shown as median with interquartile ranges. *P < 0.05; **P < 0.01; ***P <0.0001. ns, not significant. Abbreviations: AD, acute decompensation; ACLF, acute-on-chronic liver failure; IND, indeterminate; BAC, bacterial infection; GIB, gastrointestinal bleeding; ALC, active alcoholism; CPT, Child-Pugh-Turcotte score; MELD, Model for End-stage Liver Disease score; MELD-Na, Model for End-stage Liver Disease-Sodium; CLIF-C, Chronic Liver Failure Consortium; OF, organ failure; ALBI, albumin-bilirubin grade. (TIFF) [file pone.0250062.s004.tiff]

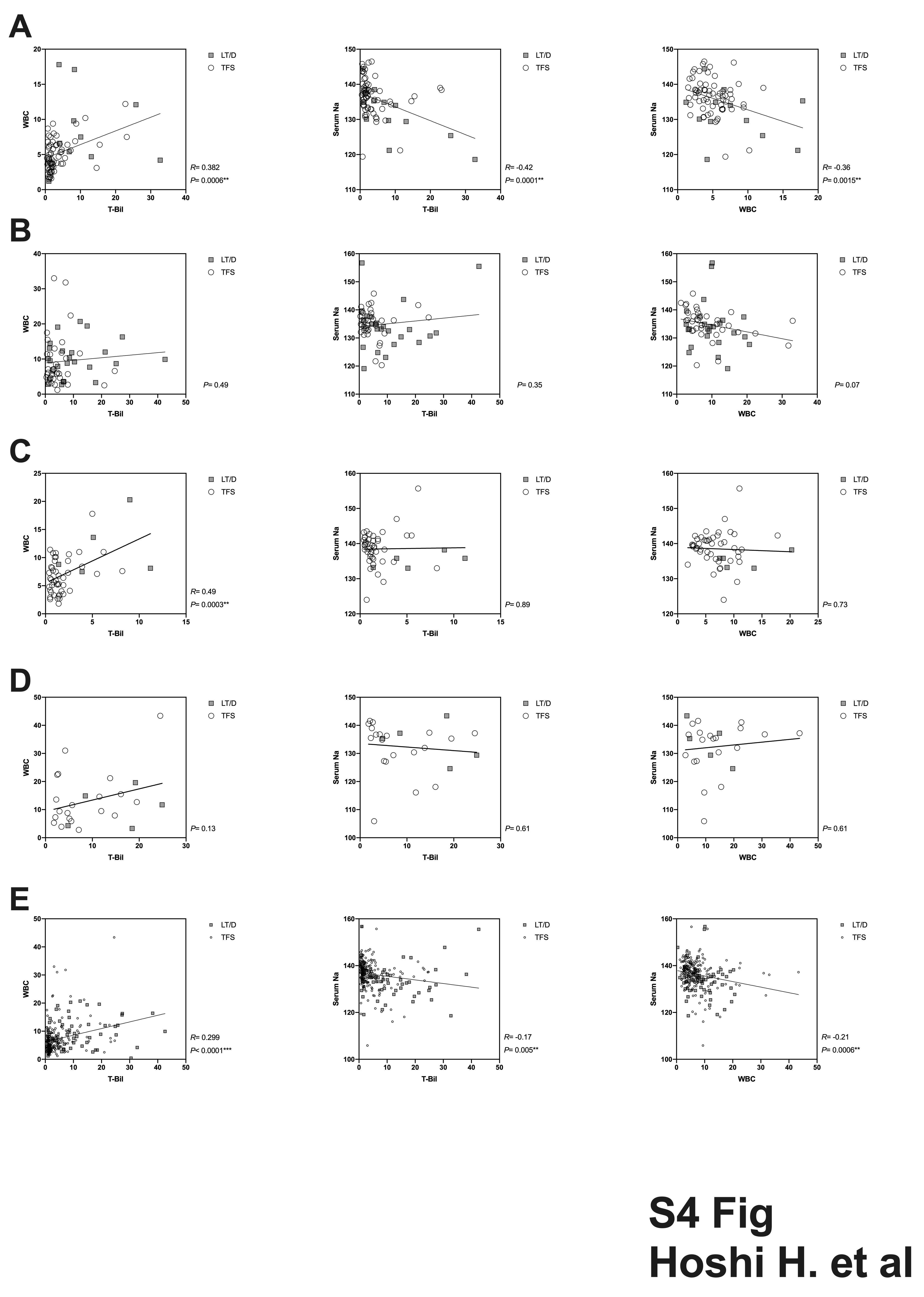

Supplement: S4 Fig — Spearman’s correlation analysis of clinical parameters in patients with their first indexed AD/ACLF stratified acute precipitants: Indeterminate (panel A), bacterial infection (panel B), gastrointestinal bleeding (panel C), alcoholism (panel D), and the whole cohort (panel E), are shown. Correlation coefficients (R) are shown if statistically significant. Open circles, transplant-free survival; shaded squares, liver transplanted/died. Units: T-bil, in mg/dL; Na, in mEq/L; WBC, in x109/L. *P < 0.05; **P < 0.01. Abbreviations: T-bil, total bilirubin; WBC, white blood cell; AD, acute decompensation; ACLF, acute-on-chronic liver failure. (TIFF) [file pone.0250062.s005.tiff]

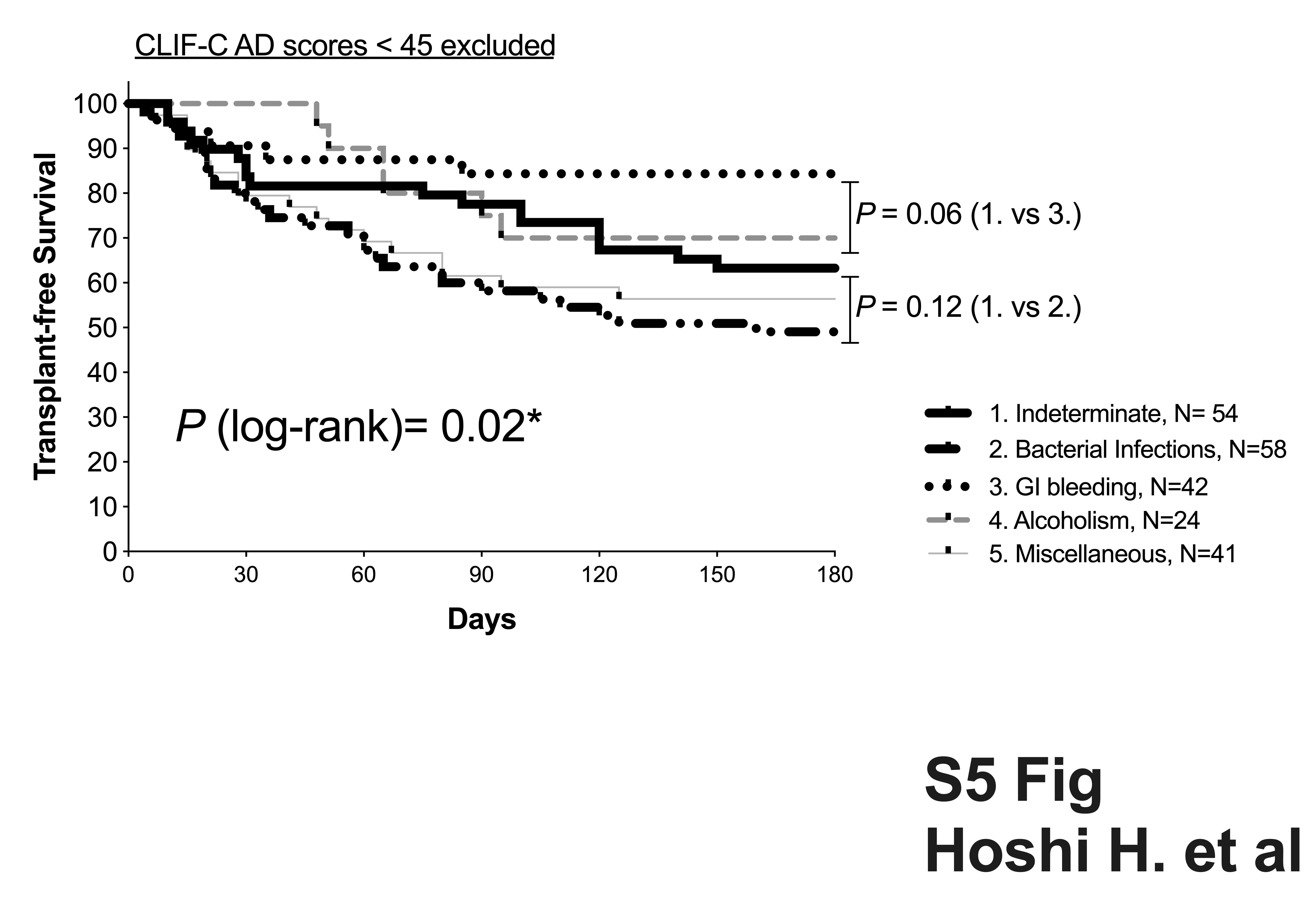

Supplement: S5 Fig — (TIFF) [file pone.0250062.s006.tiff]
