# Supplementary material for: Vulnerability to recurrent episodes of acute decompensation/acute-on-chronic liver failure characterizes those triggered by indeterminate precipitants in patients with liver cirrhosis
Source: PLoS One. 2021 Apr 13;16(4):e0250062. doi: 10.1371/journal.pone.0250062 (PMC8043384; doi:10.1371/journal.pone.0250062)
Supplement: S1 Table — (DOCX) [file pone.0250062.s007.docx]

**S1 Table. Statistical significance and AUROC of clinical parameters as possible predictors for 90-day transplant-free survival**

| ***P*/AUROC** | **1. Indeterminate** | **2. Bacterial infection** | **3. GI Bleeding** | **4. Alcoholism** |
| --- | --- | --- | --- | --- |
| **Male sex** | *P*= 0.59 | *P*= 0.59 | *P*= 0.14 | *P=* 0.06 |
| **Age over 60yrs** | *P*= 0.45 | *P*= 0.62 | *P*= 0.61 | *P=* 1.00 |
| **T-Bil, mg/dL** | ***P*= 0.005****  **AUROC=0.73**  **Cut off 4.0** | ***P*= 0.006****  **AUROC=0.70**  **Cut off 6.0** | ***P*= 0.001****  **AUROC=0.89**  **Cut off 2.6** | *P*= 0.05  AUROC= 0.78  Cut-off 18.5 |
| **Albumin, g/dL** | *P*=0.76 | *P*=0.06 | ***P*=0.0115***  **AUROC=0.81**  **Cut off 2.6** | *P=* 0.95 |
| **Serum Na, mEq/L** | ***P*=0.005****  **AUROC=0.74**  **Cut off 135.0** | *P*=0.52 | *P*=0.58 | *P=* 0.65 |
| **WBC count,10^9^/L** | ***P*=0.007****  **AUROC=0.65**  **Cut off 9.8** | *P*=0.62 | ***P*=0.011***  **AUROC=0.81**  **Cut off 7.3** | *P=* 0.53 |

**P* < 0.05; ***P* < 0.01.

Abbreviations: AUROC, Area under receiver operating characteristic; T-Bil, total bilirubin; WBC, white blood cell.
