# Supplementary material for: Vulnerability to recurrent episodes of acute decompensation/acute-on-chronic liver failure characterizes those triggered by indeterminate precipitants in patients with liver cirrhosis
Source: PLoS One. 2021 Apr 13;16(4):e0250062. doi: 10.1371/journal.pone.0250062 (PMC8043384; doi:10.1371/journal.pone.0250062)
Supplement: Attachment — Submitted filename: Response to reviewers.docx [file pone.0250062.s008.docx]

**Point-by-point Reply**

PONE-D-21-05271
Vulnerability to recurrent episodes of acute decompensation/ acute-on-chronic liver failure characterizes those triggered by indeterminate precipitants in patients with liver cirrhosis

2. The local institutional review board approved this observational study (No. 20170202) according to the guidelines of the 1975 Declaration of Helsinki (2013 revision). All study participants were adults and received standard care and treatment according to their clinical presentations. All persons gave their verbal informed consent prior to their inclusion in the study. The local institutional review board waived the need for written consent for its retrospective and observational nature.".

Please amend your current ethics statement to include the full name of the ethics committee/institutional review board(s) that approved your specific study.

Once you have amended this/these statement(s) in the Methods section of the manuscript, please add the same text to the “Ethics Statement” field of the submission form (via “Edit Submission”).

For additional information about PLOS ONE ethical requirements for human subjects research, please refer to <http://journals.plos.org/plosone/s/submission-guidelines#loc-human-subjects-research>.

**Reply:**

We amended this information, both in the revised manuscript (**revised Page 7, Line 123**) and the Submission system. Thank you.

**Reviewer #1:**

Hoshi H and co authors in the current paper examined cirrhotic patients outcome as a function of recurrent episodes of acute decompensation (AD) and/or acute on chronic liver failure (ACLF). From their data the authors conclude that AD/ACLF of indeterminate origin: i) accounted for the most of the cases (28%); ii) had a better survival; iii) exposed patients to an increased incidence of AD/ACLF recurrence during time.

This study raises the following comments:

1) The authors pooled together the data coming from AD patient and patients with ACLF. I do not think this should be considered correct. In fact as observed in the CANONIC study several statistical differences exist between these two groups. Moreover, also changes occur among different ACLF grades. Since this research mainly includes patients with no ACLF (83% of cases) I would suggest the author to restrict their analysis and conclusions on these subjects.

**Reply:**

Thank you so much for this critical comment.

We think this is one of the most important clinical question that we want to solve with this analysis. As we elaborated in the Introduction section, the aims for this research are (1) the better characterization of acute decompensation (AD) and acute-on-chronic liver failure (ACLF) that are triggered by indeterminate acute precipitant; (2) elucidation of “re- AD.”

Because the milestone CANONIC study reported by Moreau et al. (Moreau et al. Gastroenterology 2013; <http://dx.doi.org/10.1053/j.gastro.2013.02.042>) compared patients with or without “Prior AD” with a cross-sectional study design (inclusion duration: 8 months), we think that a possible survival bias may exist between these two groups under such study design, we decided to extend our retrospective observation to 8 years, and compare all patients with their 1^st^ AD, regardless of the grading of ACLF. Along with this “longitudinal design,” the predesignated outcome for analysis was set at “90-” and “180-” day transplant-free survival, since a course of “re-AD” was also one of the aims of this study, as we already showed in the Methods. Before the analyses, we also read about a follow-up report of the CANONIC study, reported by Jalan et al. published in 2015 (Jalan R J Hepatol 2015; <https://doi.org/10.1016/j.jhep.2014.11.012>). The follow-up report noticed that, although the first report by Moreau et al. emphasized a significant survival disadvantage of “28-day mortality” between No ACLF and ACLF, some patients with AD-No ACLF still suffered from high mortality within 90 days. Therefore, Jalan et al. designed a new score called “CLIF-C AD score” to improve prognostic value of 90-day survival in these patients (AD-No ACLF). In other words, when analyzing “90-” and “180-” day TFS, the difference between AD-No ACLF and ACLF grade 1 is not as clear-cut as comparing 28-day TFS. In order to fit our aims of analysis and the predesignated outcomes (90- and 180- day TFS), we decided to combine both subjects that were categorized as AD-No ACLF and ACLF at admission.

During our analysis, the publication of another multi-center prospective observation studies called “PREDICT study,” again, based on EASL-CLIF Consortium (the same as CANONIC study), are even more convincing about the “blurred” difference between AD-No ACLF and ACLF. In the first report from the PREDICT Study, Trebicka et al. classified an AD into one the 3 sub-categories (stable decompensated [SDC], unstable decompensated [UDC], and pre-ACLF) with “5 visits (up to 12 weeks)” of observation. Some patients have been re-classified as ACLF even if their CLIF-AD points were lower than 50 (Trebicka J Hepatol 2020; <https://doi.org/10.1016/j.jhep.2020.06.013>). In the second report from the PREDICT Study, Trebicka at al. showed that in both AD-No ACLF and AD-ACLF, patients with “proven bacterial infections” or “severe alcoholic hepatitis” had a similar survival (Trebicka J et al. J Hepatol 2020; <https://doi.org/10.1016/j.jhep.2020.11.019>), which implicated that the prognostic ability could be questioned in some specific setting in regard to precipitants.

In addition, a mechanistic study of blood metabolome of AD/ACLF reported by Moreau et al. (Moreau et al. J Hepatol 2020; <https://doi.org/10.1016/j.jhep.2019.11.009>) showed that “the eigenmetabolite was similar in the “non-renal”-ACLF group and the single kidney failure/ dysfunction group,” which implicated that the original ACLF grading (Moreau et al. Gastroenterology 2013) which defines “single non-renal organ failure” into “No ACLF,” and “single renal failure” into “ACLF grade 1” may not be explained mechanistically. Again, many following reports support that the difference between AD-No ACLF and ACLF grade 1 seems not as clear-cut as Moreau et al. concluded in 2013.

We did a re-analysis. We focus on the CLIF-AD scores, as we presented in Fig S3B. In our study, the average of whole-cohort CLIF-C AD score was 51.5 (ranges 25.7 to 100.7) at admission, and over 75% of those were above 45, a cut-off that suggests a favorable 3-months survival according to the report by Jalan R et al. (Jalan R J Hepatol 2015; <https://doi.org/10.1016/j.jhep.2014.11.012>). CLIF-C AD scores stratified by acute precipitant are also shown in Fig S3B (and we revised this panel to add a line at 45 for clarity). In other words, over 75% of our included study subject are thought to have a substantially higher mortality, especially when we follow them for 180 days. When we exclude cases of AD-No ACLF with CLIF-C AD scores below 45, we still observed a tendency that AD/ACLF_BAC_ has a worse survival outcome, followed by AD/ACLF_IND_ (**Fig_A_r1**).

**Fig_A_r1**

Therefore, we revised our manuscript, and we added a thorough elaboration of the rationale of pooling both AD-No ACLF and ACLF into analyses in the current study in the Discussion Section (**revised Page 23-24, Lines 396-414**), and added Fig_A_r1 as a supporting data (**newly added Fig S5**). We really think that the EASL-CLIF-C model of considering AD-ACLF as a functional sequence is really a great break-through of the study of natural history of liver cirrhosis, even though cautions of explanation with different time scales are necessary. Because of the reasons elaborated above, your re-consideration about this point of view is so much appreciated. Thank you.

2) The prevalence of an indeterminate cause for decompensation was higher in the CANONIC study (58.9% no ACLF, 43.6% ACLF) in comparison with what observed by the authors (28% pooled). This finding however was not commented or explained in the discussion. Please comment

**Reply:**

Thank you so much for this comment.

In our study, the prevalence of an indeterminate cause for acute decompensation was 28.8% in AD-No ACLF, and 22.2% in ACLF. As you noticed that these were fewer than the CANONIC Study. In the PREDICT Study held by EASL-CLIF-C, the prevalence turned to 61.81% in AD-No ACLF, and 29.21% in ACLF (Trebicka J et al. J Hepatol 2020; <https://doi.org/10.1016/j.jhep.2020.11.019>). As we mentioned in the introduction, indeterminate precipitant exists around 10 to 50% of various studies. Since the exact numbers also varied a little between CANONIC Study and PREDICT study, the exact reasons for the difference between our study should be difficult to explain and might be multifactorial. In **Table_A_r1**, we summarized some other major reports regarding ACLF. Of course, the criteria of ACLF differed among regions, and the prevalence of chronic liver diseases also differed geographically. We noticed a fewer percentage of indeterminate precipitants when the APASL criteria of ACLF were applied (in studies from APASL AARC or Japan; at the same time, using case-reporting collections), while a higher percentage in studies using EASL criteria, even in a study from China (Shi et al. Hepatology 2015; 10.1002/hep.27795), where HBV was most prevalent. Similar to our current study, this report from Shi et al. was also a retrospective analysis in a single center using the concept of AD-ACLF sequence. The percentage of indeterminate precipitant was 20.4% in this study, which was similar to ours (22.2% in ACLF).

**Table_A_r1**

| **Acute Precipitants**  Numbers (%) | **Europe (CANONIC)**  Gastroenterology  2013 | **APASL**  **(AARC)**  Hepatol Int  2017 | **China**  Hepatology  2015 | **Japan**  Hepatol Res  2018 |
| --- | --- | --- | --- | --- |
| Total N | 303 | 1402 | 405 | 109 |
| Study design | Prospective | Prospective | Retrospective | Retrospective |
| HBV exacerbation | - | 185 (13.2%) | 145 (35.8%) | 6 (5.5%) |
| Bacterial Infection | 98 (32.6%) | - | 113 (27.9%) | 26 (23.9%) |
| GI Bleeding | 40 (13.2%) | - | 40 (9.8%) | 7 (6.4%) |
| Alcoholism | 69 (24.5%) | 705 (50.3) | 25 (6.1%) | 50 (45.9%) |
| Others | 25 (8.6%) | HEV 9.4%  DILI 9.3%  AIH 3.9% | 9 (2%) | 8 (7.3%) |
| **Indeterminate** | **126 (43.6%)** | **67 (4.5%)** | **83 (20.4%)** | **12 (11.0%)** |
| Multiple | 39 (13.5%) | - | 36 (8.9%) | - |

DOIs for Ref:

Gastroenterology 2013; <http://dx.doi.org/10.1053/j.gastro.2013.02.042>

Hepatol Int 2017; 10.1007/s12072-017-9816-z

Hepatology 2015; 10.1002/hep.27795

Hepatol Res 2018; 10.1111/hepr.13064

Therefore, we modified our manuscript, and added a new paragraph commenting on the differences in the prevalence of indeterminate precipitants in various studies (**revised Page 26-27, Line 477-492**).

3) I suspect that the episodes of AD/ACLFIND observed by the authors mainly include cases of ascites and encephalopathy (data on this point should be reported). In this case the results observed by the authors would be probably more dependent to the prevalence of these complications (that typically have an abrupt onset, an unclear trigger, frequent recurrence and reduced mortality) in this group, rather than the occult origin of decompensation. Please comment and report data.

**Reply:**

Thank you for this valuable comment. First of all, we apologize that we carelessly omitted the presentation of details in acute decompensation (AD). We used the same definition of AD as the CANONIC Study. In the CANONIC Study, AD was defined as “the acute development of large ascites, hepatic encephalopathy, gastrointestinal hemorrhage, bacterial infection, or any combination of these” (Moreau et al. Gastroenterology 2013; <http://dx.doi.org/10.1053/j.gastro.2013.02.042>). In Supplementary Table 1 of the CANONIC Study, prevalence of each reason was shown as (total percentage over 100%, combination permitted):

In our current study, the reasons are as follows: (combination permitted)

a. Ascites, 118 (41.4%)

b. Hepatic encephalopathy, 64 (22.5%)

c. GI bleeding, 64 (22.5%)

d. Bacterial infection, 57 (20.0%)

As you commented, ascites and hepatic encephalopathy were the top two most frequent reasons of AD, both in CANONIC Study and our study. We also noticed that in a retrospective large-scale observation study with 8 years of inclusion reported by Mahmud et al., when stratified by types of AD, patients with a low grade ACLF due to ascites or hepatic encephalopathy were significantly more likely to develop a subsequent G3 ACLF compared to those with gastrointestinal bleed or infection as an AD (P < 0.001) (Fig 2B of Mahmud et al. Hepatology 2020; DOI 10.1002/hep.31012). This result may complement your comment and our finding (easier to re-AD).

We revised the method to clearly elaborate the definition (**revised Page 7, Line 134-137**), added this information to Table 1 (**revised Table 1**), and discussion in the revised manuscript (**revised Page 25, Line 448-451**).

**Minor**
Figures need to be improved

**Reply:**

Thank you for this comment.

According to the information provided by publisher, we used the Preflight Analysis and Conversion Engine (PACE) digital diagnostic tool, <https://pacev2.apexcovantage.com/> to made sure our figures met PLOS requirements. Thank you.

Reviewer #2: Chu et al conducted a single-center observational study, in which a total of 466 events of AD/ACLF in 285 patients, including their 285 first indexed AD/ACLF were analyzed to characterize the AD/ACLF that precipitated by “Indeterminate” factors. By stratified analysis of different acute precipitants, they demonstrated that the patients with AD/ACLFIND are more vulnerable to suffer from subsequent AD/ACLF and tended to developed AD/ACLF associated organ failure when compared to the AD/ACLF induced by other precipitants such as bacterial infection (AD/ACLFBAC), gastrointestinal bleeding, and active alcoholism et al. One of the major meaningful messages from this study could be that the clinicians may need apply different prognostic systems to predict the outcome of the AD/ACLF that triggered by different etiology.

The authors noticed that, although EASL CLIF-C ACLF grades (No ACLF vs. grade 1-3) significantly correlated with survival outcomes in AD/ACLFBAC, they did not significantly predict survival outcomes in AD/ACLFIND or AD/ACLFGIB or AD/ACLFALC (Fig1D).
Could the authors further deliberated the cause of it? How about using other model such as MELD.

**Reply:**

Thank you for this very important comment. We think that this is one of the facts of observation that propelled us to aim to figure out the real clinical picture of patients of AD/ACLF_IND_.

We think that there are 2 reasons that may explain this observation. One is longer observation periods; the other is the remitting and relapsing nature of AD/ACLF_IND_.

(1) longer observation periods and predesignated outcome

In the CANONIC study, high-grade ACLF (grade 2 and 3) showed significantly higher mortality within 28 days (Moreau Gastroenterology 2013; <http://dx.doi.org/10.1053/j.gastro.2013.02.042>), however, how do patients with AD-No ACLF and ACLF grade 1 who survived at the first 28 days do after 28 days was not pictured until a follow-up analysis of the CANONIC study published in 2015 (Jalan J Hepatol 2015; <https://doi.org/10.1016/j.jhep.2014.11.012>). In that study, Jalan and his colleagues showed that some patients with AD-No ACLF may still have poor prognosis within 90 days, and a novel CLIF-C AD score may predict them.

Therefore, we may deduce that “ACLF grading” is prognostic only within 28 days in patients with AD/ACLF. As we extend the period of observation to 180 days (Fig 1C and 1D), “ACLF grading” is still prognostic in AD/ACLFBAC, but not in cases precipitated by other factors.

(2) the remitting and relapsing nature of AD/ACLF_IND_

Another reason for this unexpected observation may lay in that AD/ACLF_IND_ tends to recur, even some survived from the 1^st^ AD. We used competing risk analysis to elaborate this point (Fig 4C). In Fig 4C, we observed a survival predominance of AD/ACLF_IND_ around day 70〜100, but it disappeared around day 120〜180; on the other hand, vulnerability to recurrence of AD around day 90〜180. This may explain the reason why ACLF grading of the 1^st^ AD/ACLF_IND_ cannot predict prognosis in these patients.

We tried to find out if there is a common simple parameter that may be prognostic for 180 day-transplant survival for all major acute precipitants, including MELD, as you suggested. We used the cut-offs yielded in the analyses of the whole cohort in Fig 2. Categorical kai-square analyses were used. The results are show in **Table_B_r1**. The MELD-Na score might be a promising single prognostic system for all types of acute precipitants.

**Table_B_r1**

| *P*-values | 1. Indeterminate | 2. Bacterial infection | 3. GI bleeding | 4. Alcoholism | 5. Miscellaneous |
| --- | --- | --- | --- | --- | --- |
| MELD over 18 | **0.0117*** | **0.0070**** | **0.0031**** | 0.0755 | **0.0005**** |
| **MELD-Na over 18** | **0.0151*** | **0.0305*** | **0.0001**** | **0.0222*** | **0.0003**** |
| CLIF-C OF over 8 | **0.0016**** | **<0.0001***** | 0.0990 | 0.3627 | **<0.0001***** |
| CLIF-C ACLF over 45 | **0.0115**** | **0.0057**** | 0.0600 | **0.0249*** | **0.0039**** |
| CPT over 11 | **0.0075**** | **0.0001**** | **<0.0001***** | 0.1079 | **0.0006**** |
| ALBI over 0.50 | **0.0224*** | **0.0002**** | **<0.0001***** | 0.0755 | **0.0003**** |

We revised the manuscript and added discussion about these factors (duration of predesignated outcomes and possible re-AD) (**revised Page 23-24, Line 396-414**).

In table 3, the patients with AD/ACLFBAC were associated with both leukocytosis (WBC count 7.4[4.6-11.8]) and hyperbilirubinemia (4.1[1.7-8.0]). However, in FigS4B, the Spearman’s correlation analysis of serum level of total bilirubin and white blood cell count demonstrated there is no correlation of this 2 factors with transplant-free survival and liver transplanted/dead. Is there any conflicts in at here, or with any previous publications?

**Reply:**

Thank you for this valuable comment.

In table 2, we analyzed clinical parameters and compared the differences “between” acute precipitants. Statistics demonstrating “median with interquartile ranges”, but not simply ranges. When comparing between types of precipitants, AD/ACLF_BAC_ is characterized as high T-bil and high WBC, as we showed in Table 2.

However, in Fig S4, we analyzed “correlations” between “T-bil,” “WBC,” and “serum sodium,” the three parameters that were prognostic in AD/ACLFIND (as we showed in Fig 3), within each acute precipitant. Please note that the ranges of axes X and Y are different in each panel of Fig S4. For example, in Fig S4A (AD/ACLF_IND_), axis for WBC ranges from 0 to 20 (x10^3^/mcL), while in Fig S4B (AD/ACLF_BAC_), axis for WBC ranges from 0 to 40 (x10^3^/mcL). In **Fig_B_r1**, we combined Fig S4A and S4B, to emphasize this point.

**Fig_B_r1**

Therefore, parameters that were characteristic of a specific acute precipitant, may not necessarily significantly correlate each other. We think that there is not any conflict about these points.

Because this kind of detailed analyses within each acute precipitant is unique in our study, we are sorry that we cannot find any exact previous publications supporting these points. As a known fact that WBC is a component of the CLIF-C ACLF score. We checked the original research paper (Jalan et al. J Hepatol 2014; <https://doi.org/10.1016/J.JHEP.2014.06.012>) that developed this score; however, we could find detailed data of sub-analyses regarding acute precipitants. We noticed an interesting fact that in the first report of the PREDICT Study analyzing the probability of developing ACLF from AD within 90 days, a decision tree plot was established. In this decision tree plot, WBC at a cut-off of 3000/mcL is a significantly factor in patients stratified by CRP, total bilirubin or creatinine (Trebicka J Hepatol 2020; <https://doi.org/10.1016/j.jhep.2020.06.013>). Because precipitating factors were not focused on this study, relationships between these factors are not known with the data presented. We hope that our current report may raise more investigations to address more clearly about this point in the future.

Minor issue
1. Page17. line 296, “Figs 3A-B”, should be changed to “Fig 3A-B”

**Reply:**

Thank you for pointing out this. We have modified as suggested. (**revised line 18, page 303**)

2. Page, Line 328, “(S2C Fig)”, should be changed “(Fig S2C)”

**Reply:**

Due to the format rules by this journal, we would like to keep this format as it was. Thank you.
